# Germinated brown rice and its bioactives modulate the activity of uterine cells in oophorectomised rats as evidenced by gross cytohistological and immunohistochemical changes

**DOI:** 10.1186/1472-6882-13-198

**Published:** 2013-07-30

**Authors:** Sani I Muhammad, Maznah Ismail, Rozi B Mahmud, Abubakar M Salisu, Zuki A Zakaria

**Affiliations:** 1 Laboratory of Molecular Biomedicine, Institute of Bioscience, Universiti Putra Malaysia, Serdang, Selangor 43400 UPM, Malaysia; 2 Department of Nutrition and Dietetic, Faculty of Medicine and Health Sciences, Universiti Putra Malaysia, Serdang, Selangor 43400 UPM, Malaysia; 3 Department of Radiology, Faculty of Medicine and Health Sciences, Universiti Putra Malaysia, Serdang, Selangor 43400 UPM, Malaysia; 4 Department of Veterinary Pathology, and Microbiology, Faculty of Veterinary medicine, Universiti Putra Malaysia, Serdang, Selangor 43400 UPM, Malaysia; 5 Department of pre-clinical studies, Faculty of Veterinary Medicine, Universiti Putra Malaysia, Serdang, Selangor 43400 UPM, Malaysia; 6 Department of Pharmacology and Toxicology, Faculty of Veterinary Medicine Usmanu Danfodiyo University, Sokoto state PMB 2346, Nigeria

**Keywords:** Germinated brown rice, Menopause, Uterine atrophy, Vagina dryness, Cyto-histology, Immunohistochemistry

## Abstract

**Background:**

Germinated brown rice (GBR) is gaining momentum in the area of biomedical research due to its increased use as a nutraceutical for the management of diseases. The effect of GBR on the reproductive organs of oophorectomised rats was studied using the gross, cytological, histological and immunohistochemical changes, with the aim of reducing atrophy and dryness of the genital organs in menopause.

**Methods:**

Experimental rats were divided into eight groups of six rats per group. Groups 1, 2 and 3 (sham-operated (SH), oophorectomised without treatment (OVX) and oophorectomised treated with 0.2 mg/kg oestrogen, respectively) served as the controls. The groups 4,5,6,7 and 8 were treated with 20 mg/kg Remifemin, 200 mg/kg of GBR, ASG, oryzanol and GABA, respectively. All treatments were administered orally, once daily for 8 weeks. Vaginal smear cytology was done at the 7^th^ week on all the rats. The weight and dimensions of the uterus and vagina were determined after sacrifice of the rats. Uterine and vaginal tissues were taken for histology and Immunohistochemical examinations.

**Results:**

GBR and its bioactives treated groups significantly increased the weight and length of both the uterus and the vagina when compared to Oophorectomised non-treated group (OVX-non-treated) (p < 0.05). Significant changes were observed in the ratio of cornified epithelial cells and number of leucocytes in the vaginal cytology between the oophorectomised non-treated and treated groups. There was also an increase in the luminal and glandular epithelial cells activity in the treated compared with the untreated groups histologically. Immunohistochemical staining showed specific proliferating cell nuclear antigen (PCNA) in the luminal and glandular epithelium of the treated groups, which was absent in the OVX-non-treated group. GBR improved the length and weight of the uterus and also increased the number of glandular and luminal cells epithelia of the vagina.

**Conclusion:**

GBR and its bioactives could be a potential alternative in improving reproductive system atrophy, dryness and discomfort during menopause.

## Background

The shifting trend in the management of ailments from synthetic compounds and drugs to nutraceutical and natural products has gained momentum in the last two decades. In this respect, germinated brown rice (GBR) and it bioactive compounds have been among the most widely studied, especially in the area of metabolic diseases such as obesity, cancer and diabetes [1-4]. GBR is brown rice that is soaked for 12-24 hours at varying temperature, during which it sporulates and releases its bioactive nutrients, which increase in quantity to approximately twice its original content. Some of the nutrients reported to increase include γ-amino butyric acid (GABA), dietary fibre, inositol, ferulic acid, phytic acid, tocotrinols, magnesium, potassium, zinc, γ-oryzanol and prolylendopeptidase inhibitor [5]. It has been reported that GABA increased dramatically when brown rice was soaked at 40°C in water for 8–24 hours [6]. Documented effectiveness of GBR also includes suppression of liver damage, hypercholesterolaemia and neuro-protective effects [7-9].

Menopause is a natural transition in a woman’s life in which her menstrual cycle ceases and she is no longer fertile due to depletion of ovarian follicles and a gradual decrease in ovarian production of oestrogen and other hormones [10] The decrease in oestrogen level during the menopause is associated with atrophy of the genital organs whereby the mucosa becomes inelastic, pale, dry and thin. Vaginal atrophy leads to dyspareunia, thus impairing sexual functioning, which invariably leads to psychological stress. There is also increased susceptibility to infection, trauma and urinary incontinence [11] Although conventional hormone therapy is highly effective in suppressing menopausal disorders, there is widespread concern about its safety. Previous studies reported the effects of late complications due to hormone-replacement therapy (HRT) such as breast, uterine and ovarian cancers, vaginal bloating and bleeding, and cardiovascular and other related complications [12-17].

Soy isoflavones have been established to have a selective oestrogenic effect and recent studies implicated genistein, a component of soy, in the enhancement of chemically induced colon and breast cancers [18-20].

Vaginal cytology of female rats can be used to determine the oestrous cycle, hormonal status and the relative function of the reproductive system in rats. The stages of the vaginal cell cycle correlate well with the changes in the ovaries and uterus. Also, the proportion and distribution of the various types of cells in the vaginal smear determines the healthy state of the reproductive system and the hormonal level. Proliferating cell nuclear antigen (PCNA) is as an endogenous marker for cell proliferation and has been equated to the Brdu proliferation assay [21-24]. It acts as a docking site for a number of proteins involved in the regulation of cell cycle and DNA repair [24,25]. An organ such as the uterus, which is always under the influence of ovarian hormones, serves as a good model for PCNA evaluation in proliferation [21].

The gross and histological appearances of tissues are important parameters in accessing the effect of administered compounds in both pharmacological and toxicological studies. The aim of this study is to ascertain the possibility of using germinated brown rice as a substitute to HRT in converting vaginal and uterine atrophy in menopause.

## Methods

### Experimental animals

Forty-eight mature Sprague–Dawley rats of 12 weeks of age, weighing 250–260g were procured from the Faculty of Veterinary Medicine University Putra Malaysia (Serdang, Selangor, Malaysia). They were acclimatised for 2 weeks and given feed and water adlibitum. The whole animal study was done at the animal house, Faculty of Medicine and Health Science, University Putra Malaysia where the rats were housed in plastic cages in a controlled air-conditioned room (25–30°C) with exposure to 12/12-h light/dark cycle. The study was carried out according to the guidelines for the use of animals and was approved by the Animal Care and Use Committee (ACUC) of the Faculty of Medicine with approval number UPM/FPSK/PADS/UUH/F01. After acclimatisation, the rats were grouped into eight groups of six rats each.

### GBR, drugs and chemicals

Brown rice was from Bernas Rice company (Malaysia), Cimicifuga racemosa (Remifemin® 20 mg/tab) was purchased from Schaper & Brummer GmbH&Co. (Salzgitter, Germany). Conjugated oestrogen (Premarin® 0.625 mg/tab) was procured from Wyeth Ireland Newbridge, (Co. Kildare, Ireland). Xylazine HCL 20 mg/ml and ketamine HCL were from TROY laboratories PTY Ltd (Smithfield, Australia).

### Extract and drug preparation

Brown rice phenolics were extracted using 80% methanol and analysed by HPLC as earlier reported [26], GABA was extracted from GBR and subsequently analysed using HPLC-DAD (Agilent, Santa Clara, CA) [27], oryzanol was analysed using the method reported by Azlan [28] and ASG was extracted using a combination of methanol and chloroform and quantified using GCMS [29].The four different samples were dissolved in distilled water (100 mg/5mls) using (0.2%) tween 20, homogenised with a high-speed homogeniser and a final effective dose of 200 mg/kg/day was administered orally.

Premarin(0.625 mg/tablet)was ground into powder using a blender and dissolved in distilled water (17.5 mg in 72.8 mls) making a concentration of 0.24 mg/ml, and a final dose of 0.2 mg/kg/day was administered orally [30].

Remifemin (20 mg/tablet) were ground into powder using an electric blender, dissolved in distilled water at a concentration of 1 mg/ml, and a final dose of 20 mg/kg/day was administered orally.

### Surgical procedure

The group 1rats were sham operated by exposing the ovaries and returning them back to their anatomical position. Bilateral oophorectomy was performed on all the rats in groups 2-8under anaesthesia using 12 mg/80 mg/kg xylazine/ketamine. The dorso-lateral part of the abdominal region was scrubbed and shaved, disinfected with poviden-iodine using gauze bandage. Two incisions of 1 cm long were performed on each of the caudal parts of the dorso-lateral area of the rats using sharp scissors after lifting the skin with a tom-forceps. Muscles were separated along their fibres and ovaries were located, double ligated and removed. The wound was then closed in two layers.

### Grouping and dosing

Two weeks after the surgery, treatment for the study was commenced on the rats. The group 2 rats were oophorectomised without treatment; groups 3 and 4 were treated with 0.2 mg/kg oestrogen and 20 mg/kg Remifemin, respectively, while groups 5, 6, 7 and 8 were treated with GBR, ASG, GABA and oryzanol at the dose rate of 200 mg/kg, respectively. All treatments were administered orally once a day for 8 weeks.

### Vaginal cytology

At the seventh week, vaginal smears were taken from each rat in the groups at approximately the same time of the day between 8 am-10 am over a period of four days, to minimise the incidence of transitional and missed stages. Lavage with saline (0.9% NaCl) was done by flushing the cells from the vaginal lining by introducing 0.2mls of normal saline into the vagina using a pipette and gently aspirating the content. This releasing and aspirating is done 2–3 times, then one or two drops of the resulting cell suspension is placed on a clean slide and covered with a slip and then mounted on a light microscope for viewing.

### Sacrifice, tissue sectioning and histology

At the eighth week, all the rats were sacrificed under ether anaesthesia, the uterus gently removed and the length and weight recorded immediately. They were then transferred immediately into RCL2 and stored at -80°C until analysis. The samples were later fixed in 10% formalin for 24 hours, sectioned and stained using Haematoxylin and Eosin (H&E) stain and observed under a light microscope.

### Immuno-peroxidase

Immunohistochemistry to detect the presence of PCNA was done following the same procedure reported earlier [31] with some modifications. Sections were mounted on gelatin-coated glass slides, deparaffinised in three changes of xylol and rehydrated in graded alcohol then washed with distilled water. The sections were then placed in 10 mmol citrate buffer, pH 6.0, for 10 mins at 50 W in a microwave, cooled at room temperature for 5 minutes. Non-specific bindings were covered using 5% bovine serum albumin (BSA), Sections were then incubated using H_2_O_2_ (3%) for 30 minutes to block endogenous peroxidase activity and washed in PBS_T_ and distilled water. PC10 monoclonal antibody procured from DakoCytomation (Denmark) was used as the primary antibody for an hour at a ratio of 1:200 then rinsed in PBS and reacted with polyclonal rabbit anti-mouse as the secondary antibody for 10 minutes at room temperature. The peroxidase reactions were developed in 3,3-diaminobenzidine in chromagen solution (DAB + CHROMAGEN) and counter stained with methylene blue for 2 minutes, and finally, sections were cleared in xylene and cover-slipped for examination under a light microscope.

### Statistical analysis

All samples were analysed in triplicate. Data were presented as mean ± standard deviation (SD). Differences were evaluated by one-way analysis of variance (ANOVA) and mean comparison was determined by Turkey Kramer LST, using JMP10 statistical software. Differences were considered significant at p < 0.05.

## Results

### Uterine weight and length

Grossly, the uteri of the oophorectomised non-treated control rats were atrophied compared to the sham-operated control and the oophorectomised treated rats as shown in Table 1. Significant differences were observed in the weight and length of the uterus and vagina of the oophorectomised treated group, the oophorectomised non-treated and the group treated with Remifemin (p < 0.0001). The significance levels between the groups were indicated in Table 1.

**Table 1:** Mean ± SD uterus and vaginal weight and length after 8 weeks of intervention with GBR and its bioactives in oophorectomised rats

**Groups**	**Sham**	**OVX**	**OVX + oestrogen 0.2 mg/kg**	**OVX + Remifemin 20 mg/kg**	**OVX + GBR 200 mg/kg**	**OVX + ASG 200 mg/kg**	**OVX + ORZ 200 mg/kg**	**OVX + GABA 200 mg/kg**
Uterus weight(g)	0.91 ± 0.18^b^	0.41 ± 0.08^c^	1.31 ± 0.32^a^	0.56 ± 0.05^c^	0.85 ± 0.04^b^	0.88 ± 0.07^b^	0.89 ± 0.06^b^	0.87 ± 0.04^b^
Uterus length (cm)	5.05 ± 0.39^a^	3.21 ± 0.41^b^	5.85 ± 0.89 ^a^	3.99 ± 0.31^b^	5.06 ± 0.44 ^a^	5.21 ± 0.40 ^a^	5.14 ± 0.35 ^a^	5.06 ± 0.29 ^a^
Vaginal weight(g)	0.19 ± 0.05^a^	0.06 ± 0.01^b^	0.17 ± 0.04^a^	0.05 ± 0.01^b^	0.18 ± 0.06^a^	0.18 ± 0.06^a^	0.19 ± 0.04^a^	0.17 ± 0.04^a^
Vaginal length (cm)	0.94 ± 0.08^a^	0.27 ± 0.06^b^	0.89 ± 0.08^a^	0.33 ± 0.03^b^	0.94 ± 0.04^a^	0.93 ± 0.04^a^	0.91 ± 0.05^a^	0.90 ± 0.07^a^

Means Comparisons for all pairs using Tukey-Kramer HSD **(**values with different superscripts a, b, c in the same row are significantly different p < 0.0001).

### Vaginal cytology

**Figure 1:**
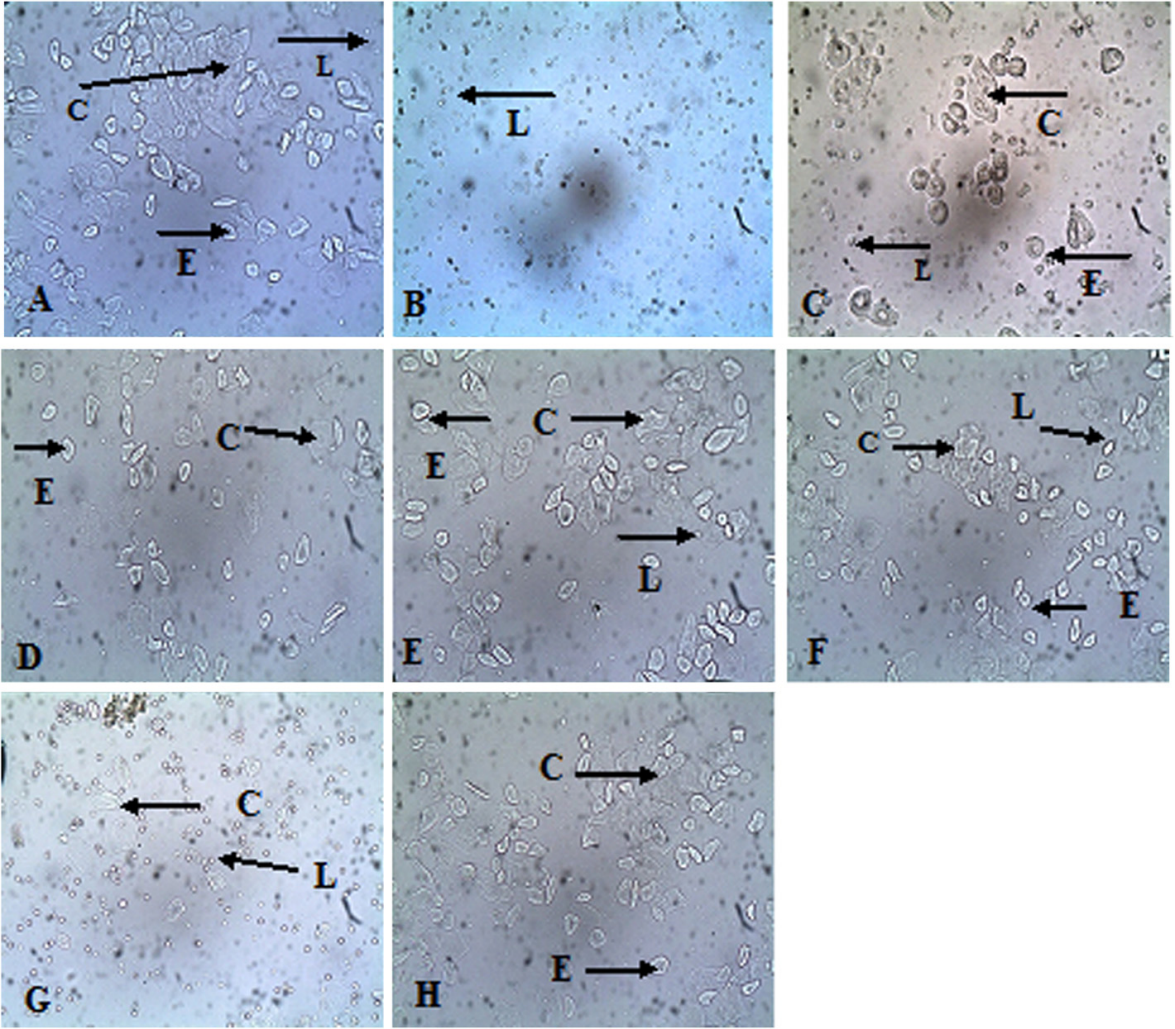
**Unstained vaginal smears (cytology) of the female rats at the 7**^**th **^**week after ovariectomy.** The diagram shows various stages of the oestrous cycle depending on the average of the dominating cells after four days of consecutive sampling in various treatment groups. (E = Epithelial cells, C = Cornified cells, L = Leucocytes). **(A)** Sham operated (No OVX), **(B)** OVX (No treatment), **(C)** oestrogen treated (OVX + EST), **(D)** ASG fraction (OVX + ASG), **(E)** GABA fraction (OVX + GABA), **(F)** GBR fraction (OVX + GBR), **(G)** Remifemin treated (OVX + REM), (**H**) (OVX + Oryzanol).

The vaginal cells (cornified and epithelial) increased in number in oophorectomised treated rats at the 7^th^ week of treatment compared to the oophorectomised non-treated group (Table 2 and Figure 1).

**Table 2:** **Proportions of vaginal cell count at the 7**^**th**^** week after treatment of oophorectomised rats with germinated brown rice and its bioactives**

**Groups cell type**	**Sham**	**OVX**	**OVX + EST 0.2 mg/kg**	**OVX + GBR 200 mg/kg**	**OVX + REM 20 mg/kg**	**OVX + ASG 200 mg/kg**	**OVX + ORZ 200 mg/kg**	**OVX + GABA 200 mg/kg**
Cornified cells	+	-	++	++	+	+	+	+++
Epithelial cells	+++	+	+++	++	+	+++	+++	+
Leucocytes	+	+++	+	+	+	+	+	+

Key: +++ = high, ++ = moderate, + = low, - = absent.

*EST* Estrogen.

*REM* Remifemin.

### Histology

**Figure 2:**
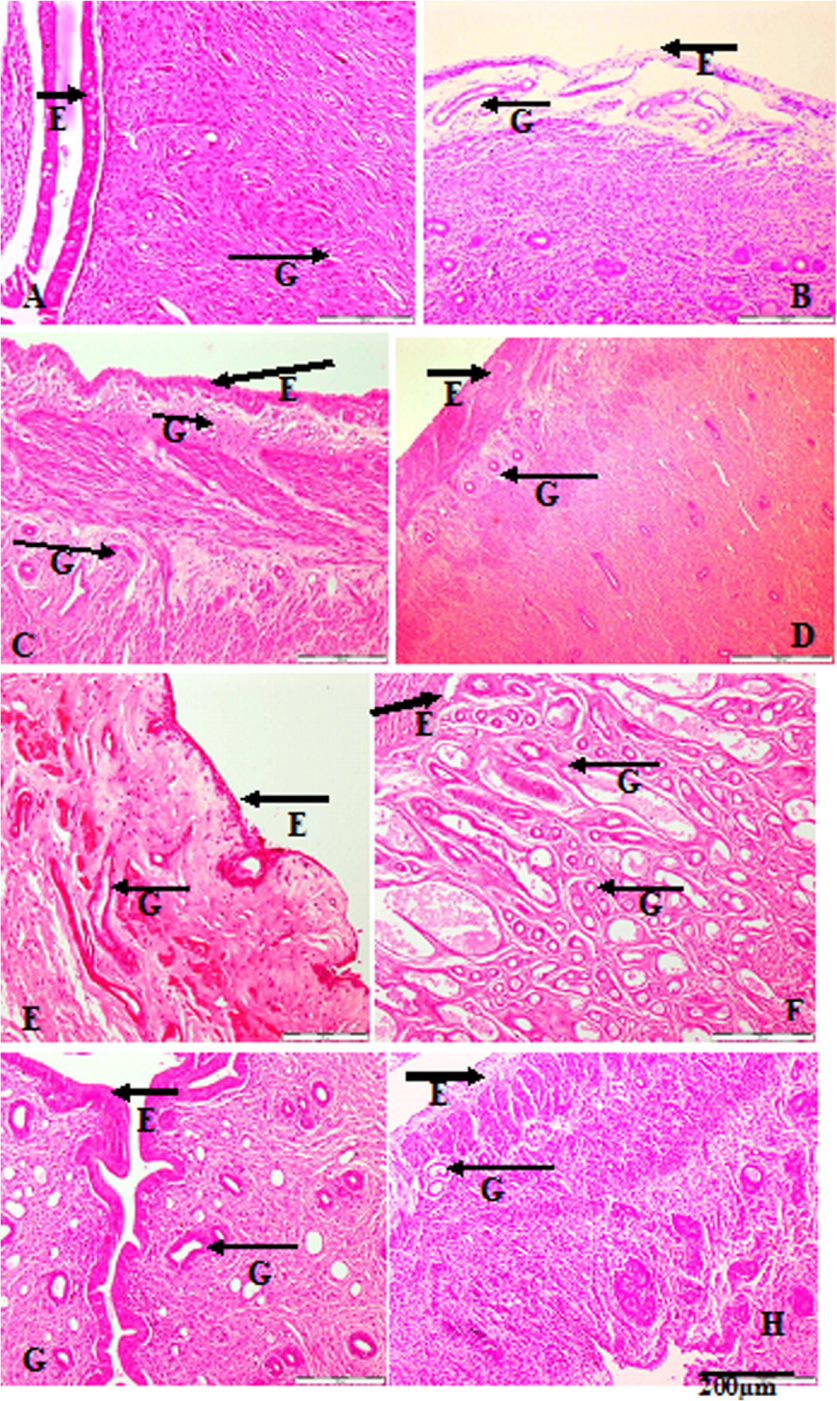
**Histological sections of oophorectomised rats uteri treated with the different fractions of the compounds for 8 weeks (H&E STAIN) ×100.** E = Epithelial cells; G = Glandular cells. **(A)** Sham operated (No OVX), **(B)** OVX (No treatment), **(C)** oestrogen treated (OVX + EST), **(D)** ASG fraction (OVX + ASG), **(E)** GABA fraction (OVX + GABA), **(F)** GBR fraction (OVX + GBR), **(G)** Remifemin treated (OVX + REM), **(H)** (OVX + Oryzanol).

Histologically, the sham-operated non-treated group showed normal uterine activity, while the oophorectomised non-treated group showed depleted epithelia with slight to absent uterine activity(Figure 2A and B). The oestrogen-treated group had an increased number of glands and epithelial lining, the group treated with ASG at 200 mg showed an increased number of glands, while the epithelia lining was slightly depleted (Figure 2C and D). The uteri of 200mgGBR-treated rats showed increased glandular formation containing fluid that showed activity and the γ-oryzanol-treated group(ORZ 200 mg) exhibited an increase in proliferation of uterine glands, which were very active (Figure 2F and H). Remifemin (20 mg) showed an increase in the number of the uterine glands and empty lumens with increased epithelial lining, The group treated with the GABA fraction (200 mg) showed an increase in uterine glands, and slight epithelial depletion (Figure 2G and E).

### Immunohistochemistry

**Figure 3:**
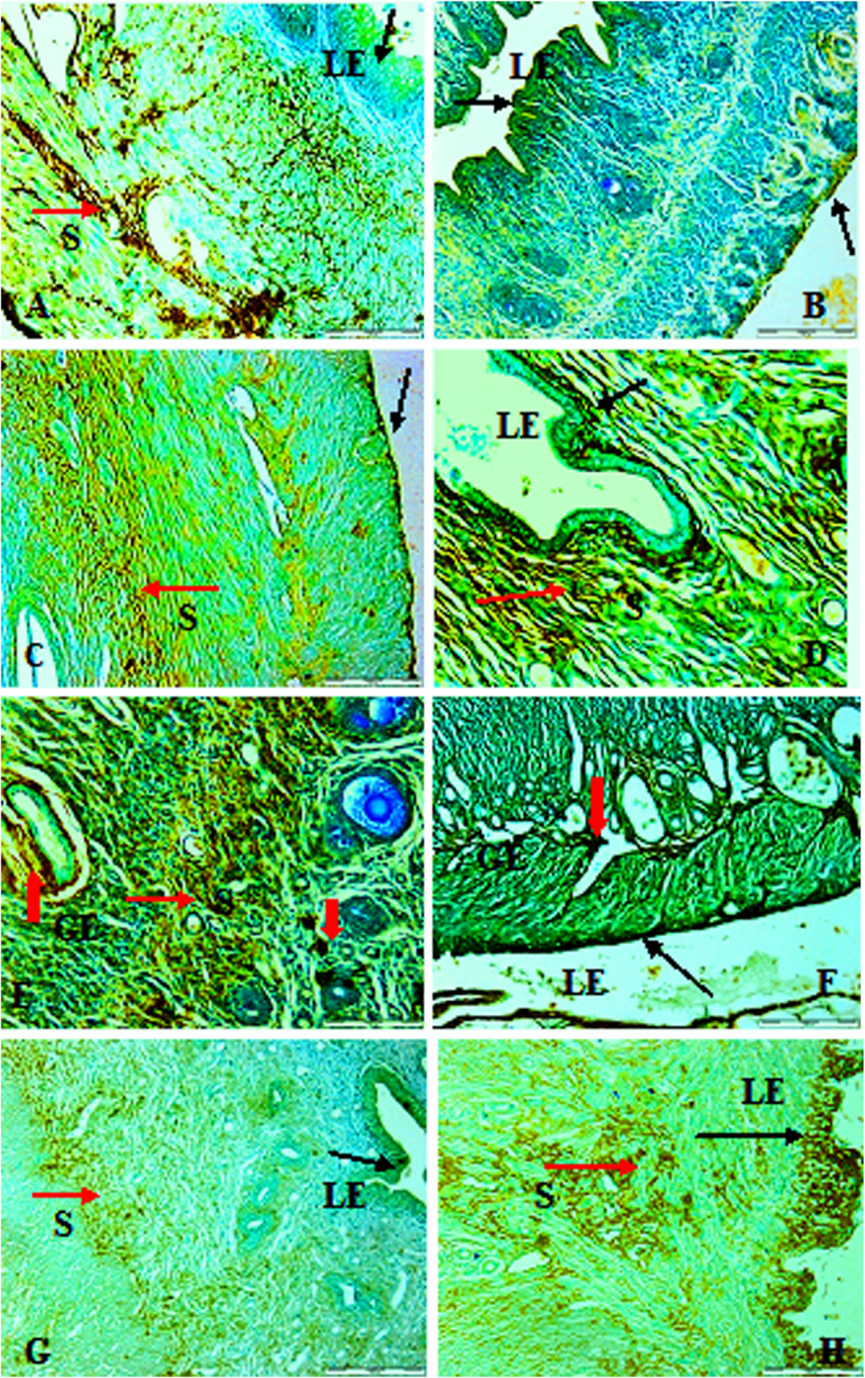
**Immunohistochemical staining of PCNA showing specific sites of binding on the nuclei in the luminal, glandular epithelium and stromal cells of oopherectomised rat uteri treated with the different fractions of the compounds for 8 weeks.** LE = PCNA positive cells staining at the luminal epithelial cells; S = PCNA positive cells staining ofthe stromal cells; GE = PCNA positive cells staining at the glandular epithelial cells cells. **(A)** Sham operated (No OVX), **(B)** OVX (No treatment), **(C)** oestrogen treated (OVX + EST), **(D)** ASG fraction (OVX + ASG), **(E)** GABA fraction (OVX + GABA), **(F)** GBR fraction (OVX + GBR), **(G)** Remifemin treated (OVX + REM), **(H)** (OVX + Oryzanol).

Immunoreactivity for PCNA was observed in the luminal and glandular epithelium, as well as the stromal cells, which was observed in positive control sections and also in treated groups. Positive cells exhibited a dark-brown homogeneous staining, while the light-brown staining areas showed less reactivity. Sham-operated rats as well as oestrogen-, oryzanol- and Remifemin-treated groups all showed PCNA reaction on stromal and luminal epithelial linings as shown in Figures 3A, C, G and H. Groups treated with ASG, GBR and GABA showed PCNA reaction on stromal, luminal and glandular epithelial cells, with the group treated with GABA showing strong reactivity as shown in Figures 3D, E and F. Oophorectomised non-treated rats showed a mild PCNA reaction only on the columnar epithelial lining (Figure 3B).

## Discussion

The use of natural substances as hormonal-replacement therapy in menopause is well documented in the literature though the availability and safety margin is a source of concern. In this study, germinated brown rice and its bioactives after eight weeks of oral treatment grossly restored the weight and length of atrophied uteri and vagina of rats compared to those of oophorectomised non-treated control group. A significant increase in both the weight and length of the uterus and the vagina was observed when comparing the sham-operated with the oophorectomised non-treated groups, and the groups treated with GBR, ASG, oryzanol, GABA and oestrogen with the group treated with Remifemin(p < 0.0001). Similar effects were observed on uterine weight between oophorectomised and non-oophorectomised rats treated with Daidzine, Genistein, oestradiol or honey [32-34].There are observable differences on the vaginal cytology between the various treatment groups compared with the oophorectomised non-treated rats, including the ratio of distribution between the three cells. Cornified cell were observed in high number in the group treated with GABA fractions, the epithelial cells were observed in higher number in groups treated with oestrogen, oryzanol and ASG, while the group treated with GBR had almost an equal number of the two cells. Oestrous cycle is a rhythmic reproductive cycle that is influenced by the release of gonadotropin-releasing hormone from the hypothalamus, gonadotropins from the pituitary gland and sex hormones from the gonads [35]. The average number of vaginal cells at a particular time of the oestrous cycle gives a clear picture of the functional status of the neuro-endocrine – reproductive system and ovarian activity [36]. Therefore, the high number of cornified as well as epithelial cells observed after treating the rats with GBR and its bioactives indicate the effects of these compounds in restoring the neuro-endocrine reproductive and ovarian function already lost due to oophorectomy. These changes observed on vaginal cytology and gross modification may be an indication that GBR and its bioactives possess oestrogenic activity, and Remifemin has little or no effect on the uterine tissue as earlier reported [37]. Histologically, rat uteri treated with GBR and its bioactives showed increased activity of the uterine cells and also the glands. PCNA, which is an indicator of DNA synthesis as well as cell proliferation, showed positive reactions on the treated cells and this showed that the cells are on their early propagation and duplication at the synthetic (S) phase cycle as reported by other workers [38,39]. Thus, the cells of the treated groups were in the early stages of duplication and showed strongly positive nuclear reactivity. The PCNA reaction also correlated well with the stages of oestrous cycle in this study and this finding is comparable with an earlier study [21]. It has been reported that mitosis usually increased from dioestrus toward proestrus, while cell death and apoptosis was low at this stage, and high towards oestrous, and the observation is related to the concentration of ovarian steroid hormones that increases at dioestrus and proestrous and decreases towards oestrous [40,41]. In this study, we observed that our sham- and oophorectomy-treated rats hada higher average number of cornified and epithelial cells than the oophorectomised non-treated groups, which showed that the GBR- and its bioactive compound-treated oophorectomised rats were mostly at their pro- or dioestrous stage of the oestrous cycle. A positive light-brown staining immunoreactivity was observed in the control sham groupand ASG- and oryzanol-treated groups at both the stroma, glandular and luminal epithelium –this clearly indicated that the induction of proliferation by these compounds was controlled and minimal. Previous studies have shown that GBR contains a lot of bioactives that have antioxidant [26,42], anti-colon cancer [43], anti-diabetic [29,44-46] and other numerous effects, ASG, for instance, which is a glycosidase, has been implicated in physiologically important processes in plants, such as response to biotic and abiotic stresses, defence against herbivores, activation of phytohormones, lignification and cell-wall remodelling [47]. Despite the fact that the different compounds give almost the same gross effects in terms of restoring the measured anatomical indices of the uterus, cytological evidences showed variability in distribution of leucocytes and epithelial and cornified cells across the treatment groups(Table 2). Likewise, the histological appearance of the various cells in the groups showed different configurations in term of glands, epithelial lining and activity (Figure 2). Furthermore, the PCNA reaction observed within the various treatment groups varied in the staining pattern of the cells. These differences may be as a result of different molecular mechanisms of action, which need to be ascertained in further studies.

## Conclusion

The uterine modulatory effects of GBR and its bioactives observed in this study might be due to the antioxidant and anti-inflammatory effects of its various components. Studies are also underway to determine whether GBR has oestrogen receptor binding activity and other effects relating to mechanisms of the utero-trophic modulations of GBR in menopause.

## Competing interests

The authors declared no competing interest with PadiBeras Nasional Malaysia (BERNAS), which will directly or indirectly influence the findings of this study.

## Authors’ contributions

SIM, RBM and MI participated in the research design, animal modelling, anatomical measurements, vaginal cytology, drafting the manuscript and all the stages involved in the development of the study. AMS and ZAZ participated in the preparation and interpretation of the histological slides, IHC staining and its interpretations, and also in writing the manuscript and its corrections. All authors read and approved the final manuscript.

## Pre-publication history

The pre-publication history for this paper can be accessed here:

http://www.biomedcentral.com/1472-6882/13/198/prepub
